# Supplementary material for: Suppression of Linear Ubiquitination Ameliorates Cytoplasmic Aggregation of Truncated TDP-43
Source: Cells. 2022 Aug 3;11(15):2398. doi: 10.3390/cells11152398 (PMC9367985; doi:10.3390/cells11152398)
Supplement: Supplementary file 1 [file cells-11-02398-s001.zip › cells-1806013-supplementary.pdf]

Supplementary Materials for

Suppression of Linear Ubiquitination Ameliorates Cytoplasmic Aggregation of Truncated TDP-43

Qiang Zhang, Seigo Terawaki, Daisuke Oikawa, Yoshinori Okina, Yoshinosuke Usuki, Hidefumi Ito, and Fuminori Tokunaga

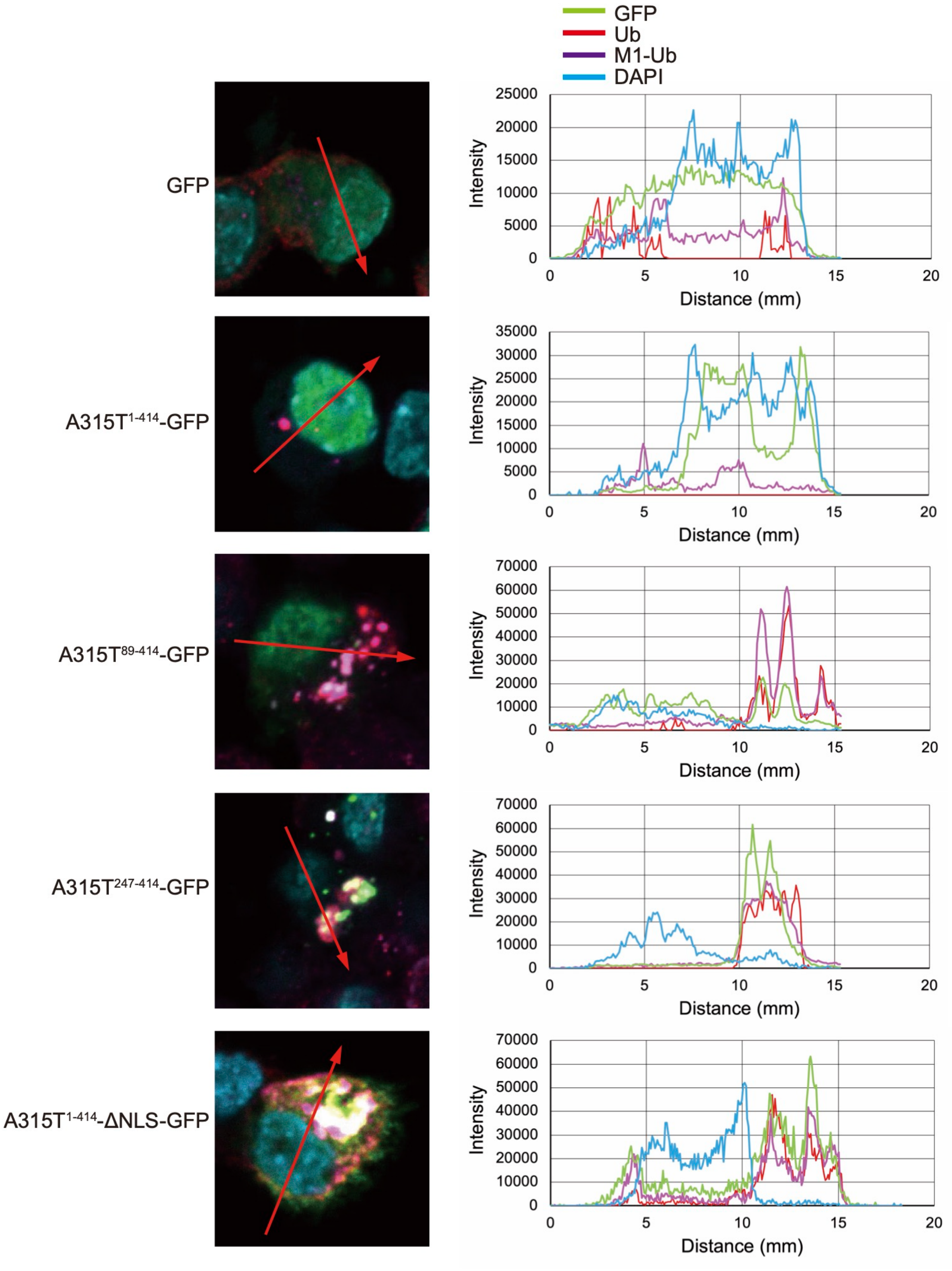

Figure S1. Colocalization of M1-ubiquitin and cytoplasmic aggregates of truncated TDP-43. Based on the immunofluorescent staining data shown in Figure 1B, intensity profile plots of GFP, ubiquitin (Ub), linear ubiquitin (M1-Ub), and DAPI signals on the *arrows* are quantified.

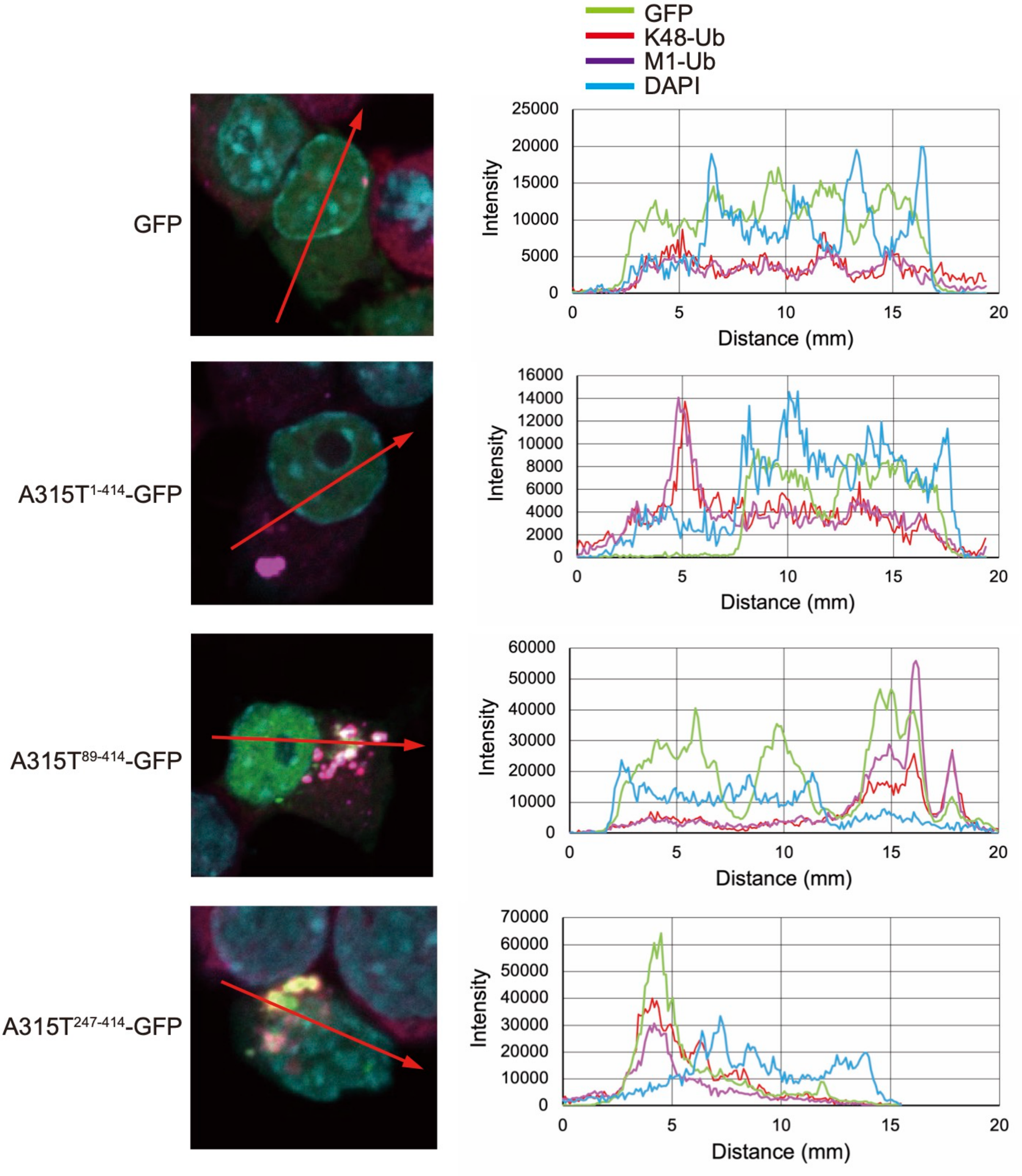

Figure S2. Colocalization of K48- and M1-ubiquitin on cytoplasmic aggregates of truncated TDP-43. Based on the immunofluorescent staining data shown in Figure 2A, intensity profile plots of GFP, K48-Ub, M1-Ub, and DAPI signals on the *arrows* are quantified.

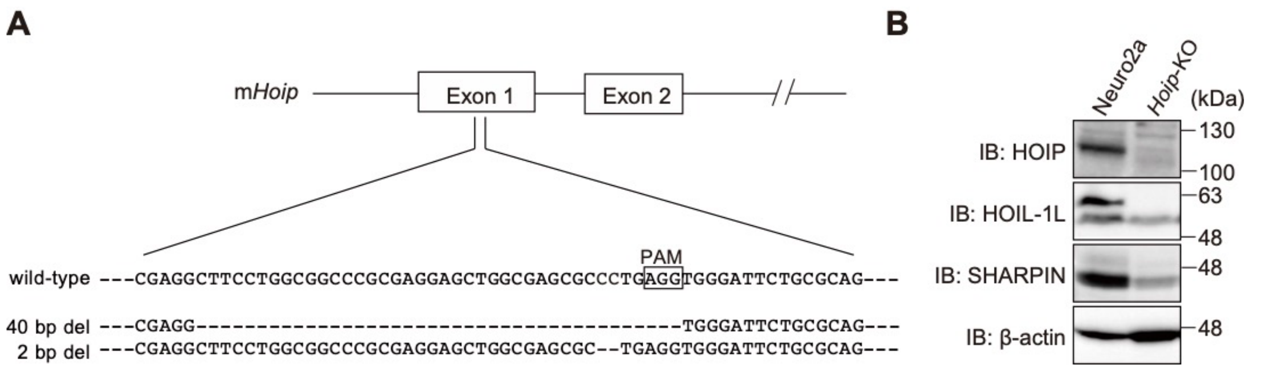

Figure S3. Construction of *Hoip*-KO Neuro2a cells. (**A**) Scheme for *Hoip*-KO targeting. The gRNA was employed to target exon 1 of the *Hoip* gene in Neuro2a cells. The nucleotide sequences of wild-type and the constructed *Hoip*-KO Neuro2a cells are shown. The PAM sequence is boxed. (**B**) Evaluation of *Hoip*-deficiency. Cell lysates from parental Neuro2a cells and *Hoip*-KO cells were immunoblotted with the indicated antibodies.

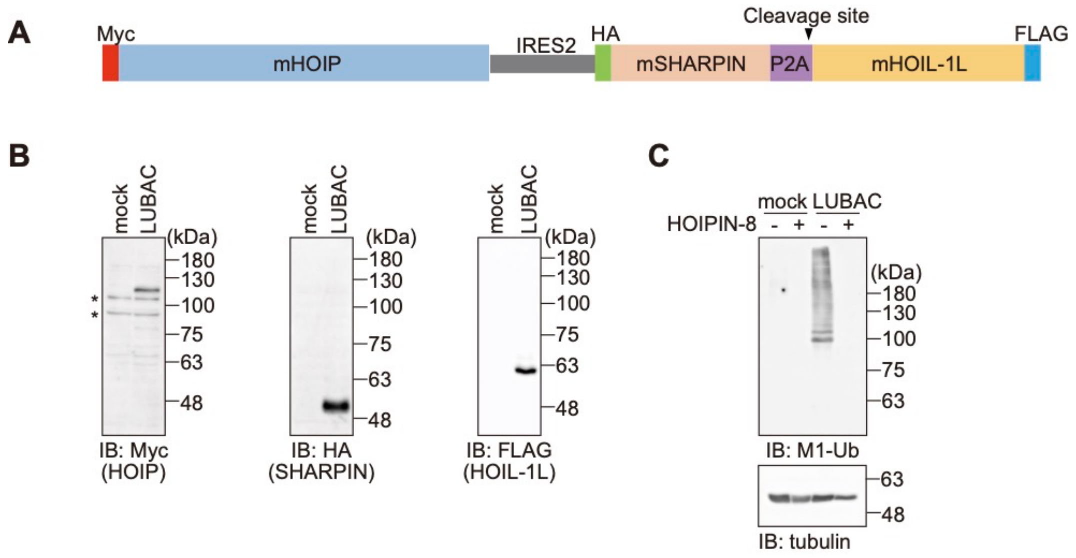

Figure S4. Simultaneous expression of LUBAC subunits. (**A**) Scheme for the expression vector tandemly encoding LUBAC subunits. (**B**) Confirmation of LUBAC expression. A mock or LUBAC-expressing vector was transfected into HEK293T cells, and the cell lysates were subjected to immunoblotting using the indicated antibodies. *: non-specific signal. (**C**) Expression of LUBAC upregulates intracellular linear ubiquitin. A mock or LUBAC-expressing vector was transfected into cells, which were then treated with 10 μM HOIPIN-8 for 20 h. The cell lysates were immunoblotted with the indicated antibodies.

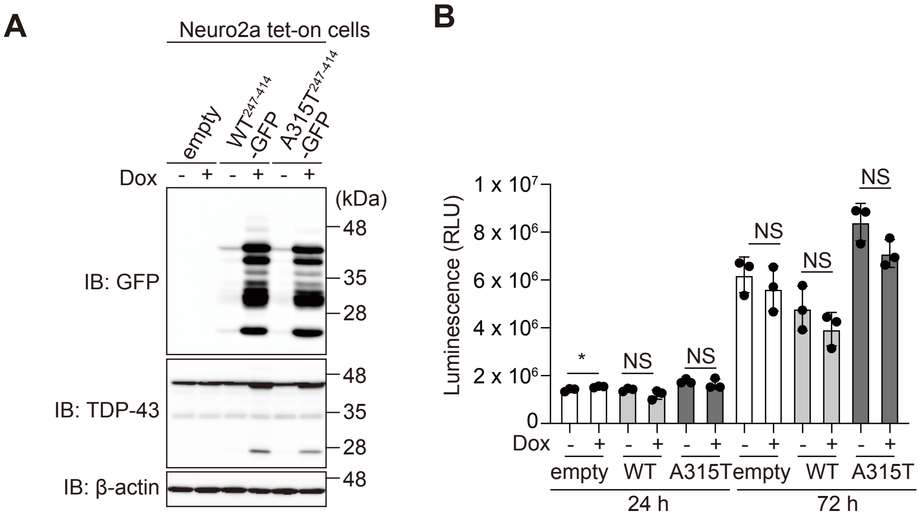

Figure S5. Construction of doxycycline (Dox)-inducible truncated TDP-43-expressing Neuro2a cells. (**A**) Induction of truncated TDP-43s. Neuro2a *tet*-on cells expressing empty vector, $WT^{247\text{-}414}$-GFP, or $A315T^{247\text{-}414}$-GFP were constructed, and cell lysates treated with or without 1 μg/ml Dox for 24 h were immunoblotted with the indicated antibodies. (**B**) Induction of truncated TDP-43s showing the minor effects on cell viability. Neuro2a *tet*-on cells expressing empty vector, $WT^{247\text{-}414}$-GFP, or $A315T^{247\text{-}414}$-GFP were cultured with or without 1 μg/ml Dox for 24 h and 72 h, and then the ATP levels were measured by using CellTiter-Glo. Data are shown as mean ± SEM, n = 3 and were evaluated by the t-test. *: P<0.05, NS: not significant.

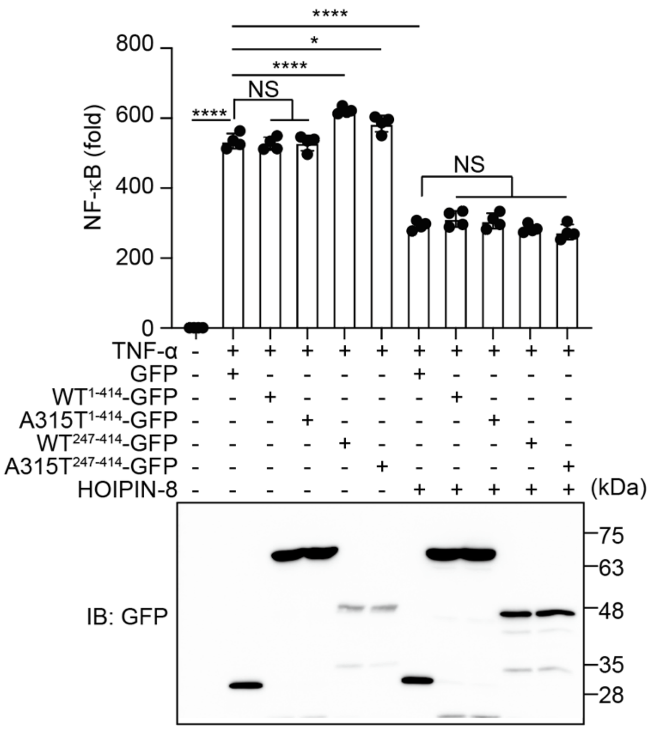

Figure S6. HOIPIN-8 suppresses the increased NF-κB activity by truncated TDP-43 in HEK293T cells. An experiment similar to that shown in Figure 7 was performed in HEK293T cells in the presence of 10 ng/ml TNF-α. Data are shown as mean ± SEM, n = 4. One-way ANOVA followed by a post-hoc Tukey HSD test. *: P<0.05, ****: P<0.0001, NS: not significant.
